# Measurement of Cancer-Related Fatigue Based on Heart Rate Variability: Observational Study

**DOI:** 10.2196/25791

**Published:** 2021-07-05

**Authors:** Chi-Huang Shih, Pai-Chien Chou, Ting-Ling Chou, Tsai-Wei Huang

**Affiliations:** 1 Department of Computer Science and Information Engineering National Chin-Yi University of Technology Taichung Taiwan; 2 Division of Thoracic Medicine Department of Internal Medicine Taipei Medical University Hospital Taipei Taiwan; 3 Division of Thoracic Medicine Department of Internal Medicine, School of Medicine Taipei Medical University Taipei Taiwan; 4 School of Nursing College of Nursing Taipei Medical University Taipei City Taiwan; 5 Cochrane Taiwan Taipei Medical University Taipei Taiwan; 6 Center for Nursing and Healthcare Research in Clinical Practice Application Wan Fang Hospital Taipei Medical University Taipei Taiwan

**Keywords:** cancer-related fatigue, heart rate variability, LF-HF ratio, photoplethysmography, wearables, chemotherapy

## Abstract

**Background:**

Cancer-related fatigue is a serious side effect of cancer, and its treatment can disrupt the quality of life of patients. Clinically, the standard method for assessing cancer-related fatigue relies on subjective experience retrieved from patient self-reports, such as the Brief Fatigue Inventory (BFI). However, most patients do not self-report their fatigue levels.

**Objective:**

In this study, we aim to develop an objective cancer-related fatigue assessment method to track and monitor fatigue in patients with cancer.

**Methods:**

In total, 12 patients with lung cancer who were undergoing chemotherapy or targeted therapy were enrolled. We developed frequency-domain parameters of heart rate variability (HRV) and BFI based on a wearable-based HRV measurement system. All patients completed the BFI-Taiwan version questionnaire and wore the device for 7 consecutive days to record HRV parameters such as low frequency (LF), high frequency (HF), and LF-HF ratio (LF-HF). Statistical analysis was used to map the correlation between subjective fatigue and objective data.

**Results:**

A moderate positive correlation was observed between the average LF-HF ratio and BFI in the sleep phase (ρ=0.86). The mapped BFI score derived by the BFI mapping method could approximate the BFI from the patient self-report. The mean absolute error rate between the subjective and objective BFI scores was 3%.

**Conclusions:**

LF-HF is highly correlated with the cancer-related fatigue experienced by patients with lung cancer undergoing chemotherapy or targeted therapy. Beyond revealing fatigue levels objectively, continuous HRV recordings through the photoplethysmography watch device and the defined parameters (LF-HF) can define the active phase and sleep phase in patients with lung cancer who undergo chemotherapy or targeted chemotherapy, allowing a deduction of their sleep patterns.

## Introduction

### Background

Fatigue is a reminder of the requirement of energy reimbursement in normal individuals. Cancer-related fatigue, defined as a multidimensional phenomenon that develops over time, is characterized by diminished energy and mental capacity and disturbed psychological conditions among patients with cancer [1]. The prevalence of cancer-related fatigue is estimated to vary from 60% to 90%, depending on the diagnostic criteria used [2]. Cancer-related fatigue often coexists with symptoms of depression, pain, anorexia, insomnia, anxiety, nausea, and dyspnea, all of which can contribute to the expression of fatigue [3]. Cancer-related fatigue imposes limitations on the normal daily activities of several patients with cancer and profoundly affects all aspects of their quality of life, including compliance with standard treatments such as chemotherapy or radiotherapy [4]. Thus, cancer-related fatigue management is an essential part of the treatment plan for patients with cancer.

To evaluate the severity of cancer-related fatigue, patients in most studies were subjectively evaluated through questions such as “How would you rate your fatigue on a scale of 0-10 over the past 7 days?” (0=no fatigue; 10=worst fatigue you can imagine). Additional measurements could be conducted using other standardized assessment tools, such as the Brief Fatigue Inventory (BFI). According to the guideline’s recommendation, regular screening of fatigue in clinical practice has been suggested [5]. However, cancer-related fatigue often remains underreported and untreated for a variety of reasons [2]. First, most of the methods used to measure cancer-related fatigue are subjective. Second, patients may gradually become accustomed to the impaired physical condition and consider discomfort as normal. Third, although several drugs have been used in clinical practice to relieve cancer-related fatigue, no standard treatments thus far have been recommended in clinical guidelines or proved effective.

Heart rate variability (HRV) is measured using a method similar to electrocardiogram (ECG) because HRV indicates the variability between two heartbeats. In practice, HRV signals are obtained from ECGs of at least 240 seconds [6] and heart rate (HR) recovery from maximal exercise testing [7]. HRV is continuously modulated through complex interactions by the autonomic nervous system, involving the sympathetic nervous system (SNS), parasympathetic nervous system (PNS), and vagus nerve [8]. SNS activities increase the HR, whereas those of the PNS reduce it [9,10]. HRV has two parameters: the time domain and frequency domain. Frequency-domain HRV is widely used to detect fatigue and its corresponding effects, such as drowsiness and sleep. Current frequency-domain measurements assign the bands of frequency into a high frequency (HF) range between 0.14 and 0.4 Hz and a low frequency (LF) range between 0.05 and 0.15 Hz. The LF-HF ratio (LF-HF) represents the relative activity between the SNS and PNS under controlled conditions [11]. A decrease in the HF band and an increase in the LF band characterize low parasympathetic activity. Previous studies have suggested that stress affects the SNS, PNS, and vagus nerve activities; thus, HRV may be used to indicate the psychological health status of patients [12,13]. HRV is widely used to measure tiredness in different scenarios and diverse disease groups [14] and has been adopted to identify the awake state and fatigue state [15]. Although not clearly understood, current evidence indicates that a higher norepinephrine level in the brain might be the core of the symptoms [16]. The HF increases during the fatigue state, whereas the LF increases during the awake state. In the non–rapid eye movement (non-REM) state, the LF-HF ratio gradually decreases as sleep deepens [15,17]. The LH-HF ratio reflects sleep activity. LF-HF differentiates the non-REM sleep from the REM state. Sleep quality at night and rest frequency during daytime can potentially correspond to a fatigue status. A higher fatigue level may cause a higher resting frequency during the daytime, affecting sleep quality at night.

Sleep activity consists of REM and non-REM states. The non-REM state consists of stages 1, 2, 3, and 4, in which a higher stage implies deeper sleep. Dreams typically occur during the REM state, and the brain becomes more active than in the non-REM state. A sleeping cycle initiates with the non-REM state, starting from non-REM stages 1-4 and back to non-REM stage 1, followed by the REM state, before the non-REM state appears again. Each cycle lasts for approximately 90-120 minutes in an average adult [18]. However, LF-HF bursts may occur in the REM state, resulting in a much higher LF-HF ratio than in the non-REM state [19-21].

### Objectives

In this study, we aim to develop objective cancer-related fatigue criteria based on HRV. We obtained the LF-HF ratio from HRV data collected from a wearable device with photoplethysmography (PPG) sensors to achieve this goal. The wearable device was built to collect HRV signals with a predefined frequency and to calculate the LF-HF ratio. The measurement was triggered per hour within 24 hours for 7 consecutive days.

## Methods

### HRV Measurement

HRV signals were contained in the data collection process during the ECG measurement (calculated). HRV is usually recorded every 5 minutes for up to 24 hours. Thus, HRV can be calculated from a similar device if the raw data can be extracted; this device has been used for overnight monitoring of ECG and HRV in cardiovascular assessments and studies [22,23]. However, lead-based ECG and HRV devices are designed for stationary measurements but not for mobile measurements, which are required in many physiological measurements. The continuous interpulse interval can be accumulated and converted into a wave, and the R-R intervals are measured and averaged to obtain information regarding the LF and HF.

PPG is a noninvasive technology that uses a light source and a photodetector at the surface of the patient’s skin to measure the volumetric variations of blood circulation and monitor personal health conditions [24]. Among the mobile designs of PPG, the wrist-worn design has become a commercialized device for revealing general health indicators calculated from the volumetric variations of blood circulation or for research that requires these raw data from the patients for further analysis [25]. PPG signals offer an excellent substitute for ECG recordings [24]. A previous study compared the pulse rate variability obtained from PPG with HRV information collected from a classic stationary device worn by normal individuals and found significant correlations of >82% for both time and frequency features [25]. Thus, pulse rate variability indices can be used as surrogates for HRV indices.

In this study, we used a PPG smart band device developed by a local company (ViPCare, Gadgletech) to collect PPG information from the patients. The cloud system of this device converts PPG information into common HRV parameters, which can be commonly applied for diagnostic and research purposes. Each HRV measurement required 2 minutes to obtain the HR and involved two frequency-domain parameters, namely LF and HF. The converted HF and LF information was used for further statistical analysis to estimate the sympathetic and parasympathetic activities of the patients to assess their stress and fatigue [11].

### Participants

The study protocol was approved by the Joint Institutional Review Board of Taipei Medical University (approval number N201910036). By using a convenient sampling approach, patients with lung cancer who visited the thoracic medicine clinic or those admitted to the thoracic medicine ward for chemotherapy or targeted chemotherapy in the teaching hospital of the Taipei Medical University were approached for potential enrollment. The inclusion criteria were ages 20 years and the ability to wear the PPG watch device. Patients with weak consciousness or those unable to respond to the questionnaire were excluded. Finally, 12 patients were included in the study.

### Sampling Procedures

The aim and purpose of the study were explained to the patients, and written informed consent was obtained from them before the investigation. The patients were asked to fill the BFI, Taiwan version (BFI-T), at baseline and were instructed to wear the PPG watch device immediately after finishing their questionnaire. The participants wore the PPG watch device continuously for 7 days, except while bathing. They were reminded by telephone to ensure that the device was worn at all times during the research period. After 7 days, the devices were collected from the participants during their next visit or admission or returned by the courier.

### Measurement System Overview

**Figure 1:**
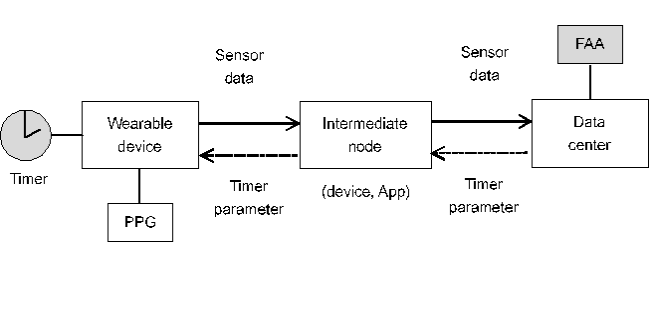
A wearable-based heart rate variability measurement system. FAA: fatigue analysis agent; PPG: photoplethysmography.

We aimed to estimate the cancer-related fatigue index based on the HRV signals of the patients. To effectively observe the changes in the cancer-related fatigue during the daily lives of the patients, we used a wearable measuring device consisting of PPG sensors to monitor the HRV signals of the patients continuously for several days (Figure 1). The device consisted of a timer that triggered PPG sensors to collect timed HRV signals transmitted to an intermediate node via Bluetooth and forwarded them to the data center through Wi-Fi or ethernet for the fatigue analysis agent for further calculations. The data center communicated with the wearables to configure the timer parameters: measurement frequency (eg, 1 hour) and duration (eg, 2 min). Cancer-related fatigue analysis procedures, such as the timed sensor data, included the HRV parameters with their timestamps. According to the timestamp, HRV parameters can be classified into different phases. In this study, we considered the active phase and sleep phase as daytime and nighttime, respectively, for the patients. The resultant parameters included the mean and variance of LF, HF, and LF-HF. The fatigue analysis agent merged the continuous HRV parameters and conducted an analysis using the BFI.

### Statistical Analysis

IBM SPSS Statistics (Version 23.0, IBM Corporation) was used for statistical analyses. Descriptive statistics, including percentage, mean, and SD, were used to present the general features of the data. Multivariate regression analysis was used to estimate the contributions of BFI to LF, HF, and LF-HF. We examined the data clusters and concerned the BFI definition (mild fatigue: <3; moderate fatigue: 4-7; severe fatigue: >7) to determine patient groups in the regressions. We used two engineering indexes, mean absolute error (MAE) and root mean square error (RMSE), to show the difference of error in paired observed values of HRV outcomes. The MAE shows the difference of error between the subjective and objective measurements whereas the RMSE represents the SD of the difference between predicted and observed values.

## Results

### Patient Characteristics

The descriptive statistics of the 12 patients are summarized in Table 1. The mean age of the patients was 65.3 years (SD 6.2), and the male:female ratio was 5:7. Most patients had a primary diagnosis of lung adenocarcinoma (10/12, 83%), and the rest had a primary cancer diagnosis of lung squamous cell carcinoma. Most patients were at an advanced stage of cancer (IV; 9/12, 75%). Targeted therapy with epidermal growth factor receptor–tyrosine kinase inhibitors was primarily used. Half of the patients were also treated with cardiovascular drugs. Most patients were also administered hypnotics to help them sleep, and most experienced some discomfort but were almost fully ambulant during the research period (Eastern Cooperative Oncology Group Performance Status=1).

**Table 1:** Descriptive statistics (N=12).

Variable		Values
Age (years), mean (SD)		65.3 (6.2)
**Sex, n (%)**		
	Female	5 (42)
	Male	7 (58)
**Primary diagnosis, n (%)**		
	Lung adenocarcinoma	10 (83)
	Lung squamous cell carcinoma	2 (17)
**Stage, n (%)**		
	Ⅰ	1 (8)
	Ⅲ	2 (17)
	Ⅳ	9 (75)
**Treatment, n (%)**		
	Target (EGFR-TKI^a^)	9 (75)
	Chemotherapy	3 (25)
**Cardiovascular drugs, n (%)**		
	No	6 (50)
	Yes	6 (50)
**Hypnotics, n (%)**		
	No	10 (83)
	Yes	2 (17)
**ECOG^b^, n (%)**		
	1	9 (75)
	2	3 (25)

^a^EGFR-TKI: epidermal growth factor receptor–tyrosine kinase inhibitor.

^b^ECOG: Eastern Cooperative Oncology Group Performance Status.

Table 2 shows the results of the subjective and objective measurements of this study obtained using the PPG watch device and self-reports of BFI-T. The results of objective outcomes included HF and LF-HF data for 7 continuous days (24 hours) during the sleep period and active period of the patients. For patients who self-reported no, mild, or moderate levels of fatigue, the frequency of LF-HF >1 during the sleep period ranged from 0% to 25%, 36.7% to 44.8%, and 80.2% to 90.7%, respectively; the mean (SD) of LF-HF ranged from 0.73 (SD 0.07) to 1.14 (SD 0.51), 1.04 (SD 0.44) to 2.12 (SD 1.65), and 2.49 (SD 1.78) to 3.19 (SD 2.29), respectively. The values of these indicators increased with the BFI-T score.

**Table 2:** Subjective and objective statistics.

Participant ID	Objective					Subjective		
	Sleeping phase			Active phase; LF-HF^a^<1 (n=14), n (%)		BFI-T^b^ score		Note
	LF-HF>1 (n=10), n (%)	LF-HF, mean (SD)						
P03	0 (0)	0.73 (0.07)	4.5 (32)		0		No	
P13	0.8 (8)	0.91 (0.44)	7 (50)		0		No	
P06	1.7 (17)	0.95 (0.20)	2.6 (19)		0		No	
P02	2.5 (25)	1.14 (0.51)	8.6 (61)		0		No	
P10	3.7 (37)	1.04 (0.44)	1.5 (11)		1.22		Mild	
P07	3.3 (33)	1.04 (0.25)	5 (36)		1.33		Mild	
P11	3.6 (36)	1.11 (0.33)	12.3 (88)		1.78		Mild	
P01	4.5 (45)	1.11 (0.27)	0.2 (1)		1.56		Mild	
P12	6 (60)	1.51 (0.73)	1.8 (13)		1.22		Mild	
P04	4.5 (45)	2.12 (1.65)	1.5 (11)		1.11		Mild	
P08	8 (80)	2.49 (1.78)	1.2 (9)		5		Moderate	
P05	9.1 (91)	3.19 (2.29)	1.9 (14)		5.67		Moderate	

^a^LF-HF: low frequency–high frequency ratio.

^b^BFI-T: Brief Fatigue Inventory-Taiwan version.

### HRV-Based Fatigue Analysis

We used HR and LF-HF for the fatigue analysis in this study. LF-HF ratios are typically around 1 in the non-REM state and are even lower for a higher non-REM stage, whereas the LF-HF ratio increases in the REM state [19,20].

**Figure 2:**
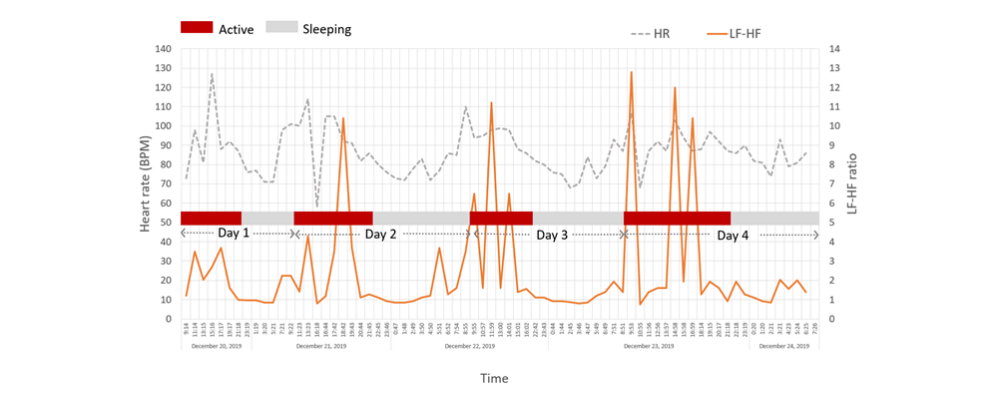
Real-time heart rate variability tracing over a duration of 4 days for patient P01. HR: heart rate; HF: high frequency; LF: low frequency; BPM: beats per minute.

Figure 2 shows the real-time HR and LF-HF ratio traces of patient P01. Typically, a high activity level leads to an increased HR and LF-HF ratio and attains a higher average HR and LF-HF ratio in the active phases. In the sleep phase, the HR decreases gradually and the LF-HF ratio decreases from >1 to <1, and accordingly, improved sleep quality. Both the HR and LF-HF ratio increased at the end of the sleep phase. Sleep events also occurred during the active phase of the patient. A low LF-HF ratio (ie, <1) with a low HR occurs on days 2 and 4. Moreover, there is a burst in the LF-HF ratio curve, which indicates an REM state in the sleep phase on day 2. HRV is monitored in a timed-measurement random sampling manner, in which HRV events are randomly sampled in a predefined interval. To precisely capture the target events, we can adopt a fine-grained measurement interval in the timer configuration. Owing to the need for a large data storage space and electric power consumption, we set the measurement time to 1 hour.

For patient *P*, we defined the LF-HF disorder ratio in the sleep phase as follows:


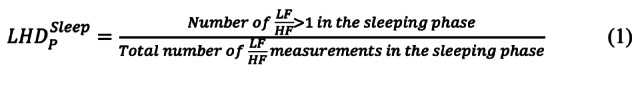


This ratio tracks the relationship between sleep quality and cancer-related fatigue. A higher value of 
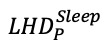
 implies a shorter deep sleep duration. The LF-HF disorder ratio in the active phase for patient *P* can be calculated as follows:


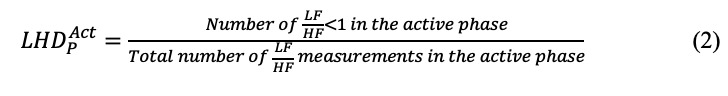


This ratio primarily tracks the fatigue level during daytime rest. A higher value of 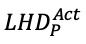 represents more frequent sleep events during daytime.

**Figure 3:**
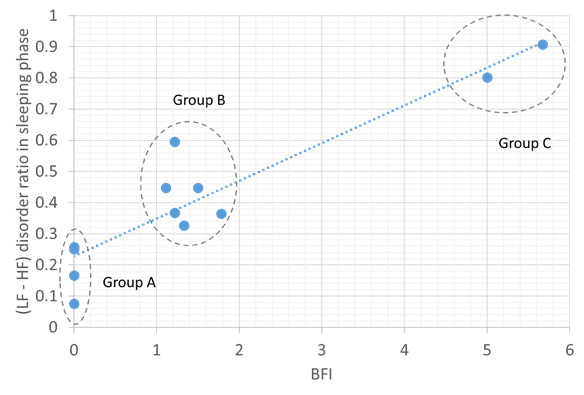
Relationship between the Brief Fatigue Inventory and low frequency or high frequency disorder ratio in the sleeping phase. The blue dotted line shows positive correlation. BFI: Brief Fatigue Inventory; HF: high frequency; LF: low frequency.

**Figure 4:**
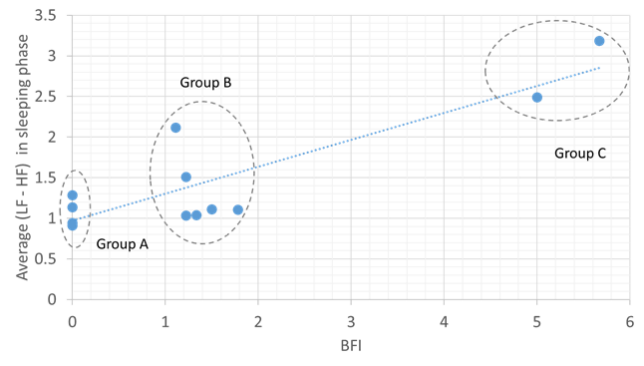
Relationship between the Brief Fatigue Inventory and average low frequency or high frequency ratio in the sleeping phase. The blue dotted line shows positive correlation. BFI: Brief Fatigue Inventory; HF: high frequency; LF: low frequency.

**Figure 5:**
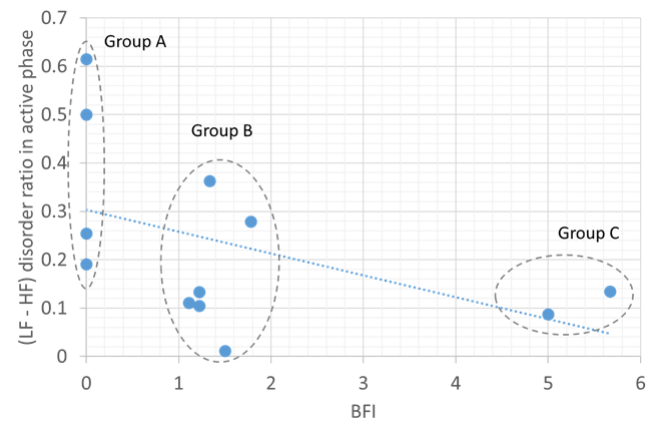
Relationship between the Brief Fatigue Inventory and low frequency or high frequency disorder ratio in the active phase. The blue dotted line shows negative correlation. BFI: Brief Fatigue Inventory; HF: high frequency; LF: low frequency.

Figures 3-5 show the correlations between objective HRV indicators and subjective BFI. Figure 3 demonstrates three facts: (1) the LF-HF disorder ratio increases with BFI in the sleep phase, (2) a strong positive correlation between the two axes (ρ=0.93), and (3) three distinct BFI groups, namely groups A (BFI=0), B (BFI=1-2), and C (BFI>3), exist between LF-HF disorder ratios of 0-1.

A moderate positive correlation was found between the average LF-HF and BFI in the sleep phase (ρ=0.86; Figure 4). The BFI groups for the LF-HF disorder ratio were distributed in a scattered manner and demonstrated a weak negative correlation in the active phase (ρ=−0.47; Figure 5). Thus, sleep quality is highly related to cancer-related fatigue, as measured by the BFI (Figures 3 and 4). Patients with high BFI (ie, high fatigue level) had poor sleep quality at night and insufficient daytime rest (Figures 3-5). The sleep quality of patients with lower BFI (ie, lower fatigue level) is generally better at night, and the resting frequency during daytime varies.

### BFI Mapping

In HRV-based BFI mapping, two HRV measurement factors need to be considered: (1) the correlation between HRV parameters and BFI and (2) the grouping effect. We defined the BFI mapping equation as follows:


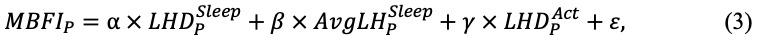


where 
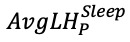
 represents the average LF-HF ratio in the sleep phase, *ε* stands for the shifting factor, and *MBFI_P_* stands for the mapped BFI for patient *P*. In equation (3), three weighting factors, namely α, β, and γ, are used to amplify or reduce the corresponding HRV parameters, depending on their correlation with the BFI, and their respective value ranges contributed to the BFI. While fitting equation (3) into a multiple linear regression model, the parameter *ε* corresponds to the intercept of the model, and based on the available data set of this study, the parameter vector {α, β, γ, *ε*} is {6.4, 0.45, 1.23, −2.07} with *R^2^*=0.87.

When calculating the mapped BFI (MBFI), the optimal vectors {α, β, γ, *ε*} for each group can be retrieved from the statistical data, and the corresponding vector varies as the data set changes. To develop a general BFI mapping method without a grouping effect, we considered a common weighting vector {α=1, β=1, γ=1} to adapt the differences observed for groups and individuals. For patient *P*, the difference between the MBFI values is shown in equation (3); the BFI is expressed as follows:


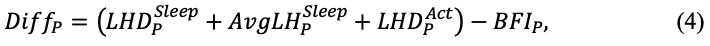


where *BFI_P_* denotes the BFI of patient *P*. If the number of patients included in group *W* is denoted as N*_W_*, the grouping compensation factor of group *W*, CF*_W_*, can be computed as


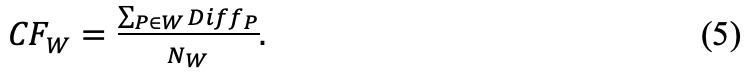


On the basis of the grouping compensation factor, the MBFI of patient *P* can be updated by rewriting equation (3), as follows:


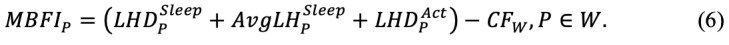


If the resultant MBFI was <0, the value of MBFI was fixed at 0.

**Figure 6:**
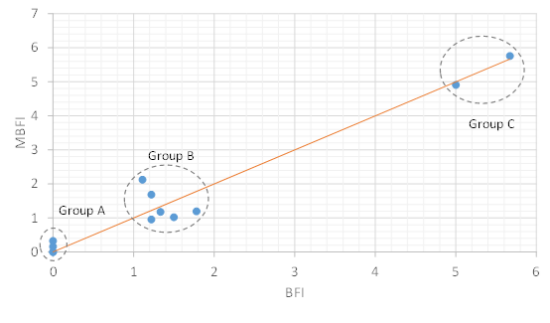
Mapped Brief Fatigue Inventory distribution. The red solid line represents mapped Brief Fatigue Inventory=Brief Fatigue Inventory. BFI: Brief Fatigue Inventory; MBFI: mapped Brief Fatigue Inventory.

Figure 6 shows the MBFI distributions for the BFIs of all patients. MBFI can be close to BFI, especially in groups A and C. For group B, the individual differences affect the approximation between MBFI and BFI in the presence of more patients. To evaluate the performance of MBFI, Table 3 presents the results of mapping errors in terms of MAE and RMSE. The MAE and RMSE are defined as follows:


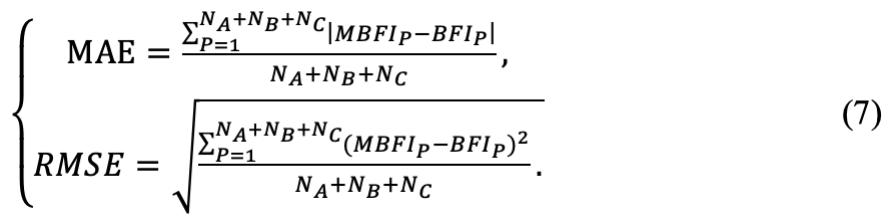


As shown in Table 3, the MAE and RMSE values for group B were approximately 0.5, and the MAE and RMSE values for groups A and C were approximately 0.1. The MAE and RMSE values of all patients were 0.3 and 0.41, respectively. As MAE is the target mapping error metric, the mapping error rate of MBFI is (0.3/10)×100%=3%, where the BFI range is 0-10. According to the resultant MAE and RMSE, the MBFI provides a fair solution for estimating the cancer-related fatigue using PPG-based HRV parameters.

**Table 3:** Mapping error statistics.

Metric	MAE^a^	RMSE^b^
Group A	0.11	0.18
Group B	0.49	0.56
Group C	0.09	0.09
Total	0.3	0.41

^a^MAE: mean absolute error.

^b^RMSE: root mean square error.

## Discussion

### Principal Findings

Analysis of the HRV data of the 12 patients with lung cancer enrolled in this study showed that the LF-HF ratio can be highly correlated with the subjective BFI, particularly that measured during sleeping time. On the basis of the correlation results, we further derived a BFI mapping equation based on the LF-HF ratio to estimate the target BFI. The analytical results showed that the total mapping error rate was 3% and that the LF-HF ratio can be considered a fair indicator to evaluate the degree of cancer-related fatigue during cancer treatment.

HRV pinpoints direct and indirect heart dynamics and respiration cycle alterations that couple differently during sleep and wakefulness across age and sex [26]. Environmental changes (eg, during the day and night) also influence HRV patterns [27]. Hence, it is not surprising that the emergence or existence of a disease and its biochemical progress affect HRV. A systematic review and meta-analysis of HRV in the prediction of cancer survival suggested that higher vagal nerve activity might predict prolonged survival [8]. HRV can be used to detect general systemic inflammation status. The Toon Health Study, which investigated 1728 nonsmoking Japanese individuals aged 30-79 years between 2009 and 2012, reported that HRV parameters are highly correlated with an elevation of C-reactive protein, a marker of inflammation [28]. HRV is probably under multicenter control, and the brain and heart are the primary control sources. Studies have provided preliminary evidence that brain damage [9,29] and heart damage [10,30] alter HRV patterns. Although the brain-heart axis mechanisms remain largely unknown, the communication between these two major organs probably involves the crosstalk of neuroimmunological chemicals [31] and induces a rise of norepinephrine in the brain that causes subsequent fatigue and sleep disturbance [16].

The current clinical measurement of HRV is based on stationary devices in a denoised environment (such as a physiological laboratory with good environmental noise isolation). Hence, only restricted activity information can be collected during the examination, which normally lasts for 5 minutes. This largely reduces the sensitivity and specificity of these hospital-based measurements. As a result, such devices are unlikely to provide an informative picture of the actual fatigue condition of patients. A comprehensive understanding of the continuous physiological changes is necessary to examine complex phenomena, such as fatigue, either for diagnostic or research purposes. In addition, a comprehensive collection of HRV information of patients with cancer enables early detection of fatigue symptoms, especially when patients fail to reveal their problems. Moreover, it helps to reduce the workload of medical staff, particularly nurses.

The main contributions of this study are the theory and application domains. In the theory domain, there are two phases in HRV data, namely the active phase and the sleep phase. Accordingly, three objective HRV parameters, namely the LF-HF disorder (LF-HF >1) ratio of the sleep phase, average LF-HF ratio of the sleep phase, and LF-HF disorder (LF-HF <1) ratio of the active phase, are related to subjective BFI results. Consequently, the MBFI can be calculated as an objective cancer-related fatigue indicator from the combination of three LF-HF-related parameters to approximate the subjective BFI.

### Limitations

This study was mainly limited by three issues. First, the sample size was relatively small and thus the power. A more rigorous design would have been to recruit a second sample to determine whether the results are generalizable. Second, because of the small sample size, we did not adjust the variance of HRV caused by the different treatments. Third, sweating on wearing the device might have caused discomfort to some patients, and others might have been worried about damaging the device during work that involved washing; thus, some data might have been lost when the patients likely removed their PPG watch device at other instances besides showering.

### Conclusions

The LF-HF ratio was highly correlated with the cancer-related fatigue. For decades, self-reported subjective assessment tools have been used to measure fatigue levels of the patients. Although the assessments may reveal the direct feelings of the patients, the exact physiological conditions could be missed owing to conservative expression and poor dysregulation of the patients. Beyond revealing fatigue levels objectively, continuous HRV recordings through the PPG watch device and the defined parameters LF-HF can outline the active phase and sleep phase in patients with lung cancer who undergo chemotherapy or target chemotherapy, allowing a deduction of their sleep patterns. Additional studies that examine the similarity and diversity of the LF-HF ratio in patients with different types of cancer are warranted.

## Abbreviations

BFI: Brief Fatigue Inventory
ECG: electrocardiogram
HF: high frequency
HR: heart rate
HRV: heart rate variability
LF: low frequency
MAE: mean absolute error
MBFI: mapped Brief Fatigue Inventory
PNS: parasympathetic nervous system
PPG: photoplethysmography
REM: rapid eye movement
RMSE: root mean square error
SNS: sympathetic nervous system
